# Safety, efficacy and biomarkers analysis of mesenchymal stromal cells therapy in ARDS: a systematic review and meta-analysis based on phase I and II RCTs

**DOI:** 10.1186/s13287-022-02956-3

**Published:** 2022-06-25

**Authors:** Jianbao Wang, Fenbin Luo, Ye Suo, Yuxin Zheng, Kaikai Chen, Deyuan You, Yuqi Liu

**Affiliations:** Department of Respiratory and Critical Care Medicine, Fujian Respiratory Medical Center, The Second Affiliated Hospital of Fujian Medical University, Quanzhou City, Fujian Province China

**Keywords:** Mesenchymal stromal cells, Acute respiratory distress syndrome, Stem cell, Mortality, Adverse events, Serious adverse events, Biomarkers, Systematic review, Meta-analysis

## Abstract

**Background:**

Mesenchymal stromal cells (MSCs) therapy for acute respiratory distress syndrome (ARDS) is an emerging treatment, but most of the current trials of MSCs stay in the animal experimental stage, and the safety and efficacy of MSCs in clinical application are not clear. We aimed to analyze the safety, efficacy and biomarkers of mesenchymal stromal cells in the treatment of ARDS.

**Methods:**

For this systematic review and meta-analysis, we searched PubMed, Embase, Cochrane Central Register of Controlled Trials, Web of science, CNKI, VIP and Wan Fang data, studies published between database inception and Mar 17, 2022. All randomized controlled trials (RCT) of stem cell interventions for ARDS were included, without language or date restrictions. We did separate meta-analyses for mortality, subjects with adverse events (AEs) and subjects with serious adverse events (SAEs). Since the trials data are dichotomous outcomes, the odds ratio (OR) is adopted for meta-analysis. The quality of the evidence was assessed with the Cochrane risk of bias tool.

**Findings:**

In total, 5 trials involving 171 patients with ARDS were included in this meta-analysis. A total of 99 individuals were randomly assigned to receive MSCs treatment, and 72 were randomly assigned to receive placebo treatment. Treatment with MSCs appeared to increase the occurrence of adverse events, but this result was not statistically significant (OR, 1.58; 95%CI, 0.64–3.91; *P* = 0.32). The occurrence of serious adverse events was lower in the MSCs group than in the placebo group (OR, 0.57; 95%CI, 0.14–2.32; *P* = 0.43); there seems to be no significant difference between the two groups in terms of 28 days mortality (OR, 0.93; 95%CI, 0.45–1.89); oxygenation index and biomarkers showed a tendency to improve in treatment, but there was a lack of more statistically significant clinical evidence to support them.

**Interpretation:**

Based on the current clinical trials, MSCs intervention has some safety for ARDS patients, but its effectiveness and predictive value of airspace biomarkers need to be determined by more large-scale, standard randomized controlled trials.

**Supplementary Information:**

The online version contains supplementary material available at 10.1186/s13287-022-02956-3.

## Introduction

ARDS is a type of acute diffuse, inflammatory lung injury [1]. The pathological features of ARDS are a diffuse alveolar injury that includes endothelial and epithelial injury in the lung [2]. Lung parenchymal injury is caused by multiple mechanisms, such as direct injury, immune-related injury, and mechanical ventilation-related injury [2]. Physiologically, refractory hypoxemia due to ventilation-blood imbalance and intrapulmonary shunting is a major characteristic of ARDS [2]. At present, the treatment of ARDS mainly focuses on mechanical ventilation and some emerging pharmacotherapy. Mechanical ventilation includes conventional small tidal volume ventilation, prone position ventilation, lung-protective ventilation, airway pressure release ventilation (APRV) and extracorporeal membrane oxygenation (ECMO) [3–6]. Compared with traditional small tidal volume ventilation, prone position ventilation and lung-protective ventilation can reduce the mortality of patients with moderate to severe ARDS, the application of early APRV can improve oxygenation and respiratory system compliance, reduce *P*_plat_ and reduce the duration of both mechanical ventilation and ICU stay [3–5]. While ECMO is mainly used in patients with severe ARDS, it is controversial whether ECMO can improve the mortality of ARDS patients [6]. Pharmacotherapies include corticosteroids, surfactants, N-acetylcysteine, statins, beta-agonists, DNase, granulocyte-colony stimulating factor (GM-CSF), human ACE2, and so on, and there is no evidence that these drugs can improve mortality in ARDS [7–9]. Although the treatment of ARDS is more comprehensive and effective with advances in medicine, the mortality rate of ARDS is still high. Based on the current state, improving mortality in ARDS patients is still an urgent need.

MSCs, as a member of stem cells, have been found to have strong immune regulation and anti-inflammatory function in the past decade in addition to proliferation and division function and are increasingly used in various diseases [10, 11]. Laffey has summarized the relevant mechanisms of action of MSCs in reviewing decades of cell therapy for ARDS: 1. Immune modulation; 2. Enhanced bacterial clearance and antimicrobial effects; 3. Injury and inflammation resolution; and 4. Restoration of capillary barrier function [2].

In recent years, lots of animal experiments have conducted in-depth research and discussion on the mechanism and efficacy of MSCs in the treatment of ARDS [12–16]. In 2017, Thomas' animal study found that MSCs improve lung injury in vivo through (extracellular vesicles) EV-mediated mitochondrial transfer promoting an anti-inflammatory and hyperphagocytic macrophage phenotype in the setting of ARDS [16]. In 2019, Lu et al. conducted a further study on the relationship between MSCs, hepatocyte growth factor (HGF) and DCregs and concluded that MSCs alleviate early ALI by inducing mature dendritic cells (MDCs) to differentiate into regulatory dendritic cells (DCs) through paracrine hepatocyte growth factor (HGF), and the mechanism of HGF-induced differentiation of mDCs into tolerogenic dendritic cells (DCregs) is related to the activation of the Akt pathway [14]. Recently, Zhang and Peng have studied the mechanism of anti-fibrotic and epithelial repair effects of MSCs, respectively, and studies have shown that high expression of epithelial–mesenchymal transition (EMT) is associated with early lung fibrosis in ARDS, mesenchymal stem cell microvesicles (MSC MVs) could inhibit the expression of EMT [13], and MSCs can also reduce the inflammatory response by inhibiting T helper cell differentiation and promoting p63 + cell proliferation and lung injury repair, the effect is associated with transcriptional inhibition of interleukin 6-phosphorylation and activation of tumor protein 63-jagged 2 signaling [12]. However, the clinical application of MSCs in the treatment of ARDS is still full of more uncertainty, and there is a lack of clear clinical evidence for both safety and efficacy. In 2020, Qu reviewed all clinical studies (including 2 cases, 3 RCTs, and 4 non-RCT studies) on MSCs for ARDS between 1990 and March 2020 to conduct a meta-analysis of safety and efficacy to propose that MSCs may have potential efficacy for COVID-19-associated ARDS, but it still needs to be confirmed by more clinical trials [17]. The aim of this study was to review all randomized controlled studies on MSCs in the treatment of ARDS as of March 17, 2022, and to conduct a comprehensive systematic review and meta-analysis of adverse events, mortality, improvement of oxygenation index, and biomarkers during MSCs treatment to fully assess the safety and efficacy of MSCs in clinical application, and to conduct a detailed evaluation of biomarkers with assessed MSCs efficacy to promote the clinical application of MSCs in the future.

## Methods

### Eligibility criteria

The inclusion criteria were as follows: (1) Population: We included randomized controlled trials of adult (age ≥ 18 years) patients with Berlin criteria-defined ARDS; (2) Interventions: Interventions were mesenchymal stromal cell, mesenchymal stem cell, stem cell, MSCs/MSC or progenitor cell; (3) Comparators: Placebo; (4) Outcomes: While each study had different outcomes measure, the main outcome measures we included were as follows: subjects with adverse events (AEs), subjects with serious adverse events (SAEs), mortality, PaO_2_/FiO_2_, ventilation‑free days to D28; and (5) Study types: randomized controlled trials (RCT), blind or not. We applied no language restrictions (Table 1).

**Table 1:** PICOS criteria and Cochrane Risk of bias in included Trails

Population	Interventions	Comparators	Outcomes	Study types
*PICOS criteria*				
Patients with Berlin criteria-defined ARDS, Age > 18 years, not animals	MSCs/MSC, Progenitor cell, mesenchymal stem cell, mesenchymal stromal cell, stem cell	Placebo	Subjects with Adverse events (AEs), subjects with Serious Adverse events (SAEs), mortality, PaO_2_/FiO_2_, Biomarkers	randomized controlled trials (RCT), blind or not

Trails	Randomization method	Allocation concealment	Blinding		Incomplete outcome data	Selective reporting	Other bias
			Participants and personnel	Assessment			
*Cochrane Risk of bias in included Trails*							
Monsel et al. [22]	Low risk	Low risk	Low risk	Low risk	Low risk	Low risk	Low risk
Bellingan et al. [21]	Low risk	Low risk	Unclear risk	Unclear risk	Low risk	Low risk	High risk
Lanzoni et al. [20]	Low risk	Unclear risk	Unclear risk	Unclear risk	Low risk	Low risk	Low risk
Matthay et al. [19]	Low risk	Low risk	Unclear risk	Unclear risk	Low risk	Low risk	High risk
Zheng et al. [18]	Unclear risk	Unclear risk	Unclear risk	Unclear risk	Low risk	Low risk	Low risk

The exclusion criteria were as follows: (1) conference papers and abstracts; (2) data cannot be extracted; (3) Not RCT; (4) clinical protocols; (5) animal; and (6) case series.

### Information sources

On March 17, 2022, we searched PubMed, Embase, Cochrane Central Register of Controlled Trials, Web of science, CNKI, VIP and Wan Fang data, studies published between database inception and Mar 17, 2022.

### Search strategy

The search strategy was to use the following terms: ARDS; Acute Respiratory Distress Syndrome; ALI; acute lung injury; Shock Lung; Respiratory Distress Syndrome; mesenchymal stromal cell; stromal cell; mesenchymal stem cell; stem cell; Mesenchymal Progenitor Cell; Progenitor Cell; MSCs/MSC; and randomized controlled trial. We also provide the specific search strategy of four English databases, and the details are shown in Additional file 1: Table 1.

### Selection process

Two researchers independently screened titles and abstracts of all articles retrieved. In case of disagreement, consensus on which articles to screen full text was reached by discussion. If necessary, the third researcher was consulted to make the final decision. Next, two researchers independently screened full-text articles for inclusion. Again, in case of disagreement, consensus was reached on inclusion or exclusion by discussion, and if necessary, the third researcher was consulted.

### Data collection process

We designed a data extraction form, which two review authors used to extract data from eligible studies. Extracted data were compared, with any discrepancies being resolved through discussion.

### Data items

Eligible outcomes were broadly categorized as follows: subjects with adverse events (AEs), subjects with serious adverse events (SAEs), mortality, PaO_2_/FiO_2_, MSCs source, administration route, dose of MSCs, biomarkers (Additional file 2: Table 2 and Additional file 3: Table 3).

### Study risk of bias assessment

We assessed risk of bias in the included studies using the revised Cochrane ‘Risk of bias’ tool for randomized trials (RoB 2.0). RoB 2.0 addresses six specific domains: (1) randomization method; (2) allocation concealment; (3) blinding of participants and personnel or assessment; (4) incomplete outcome data; (5) selective reporting; and (6) other bias. According to the results of each study, the judgment on low risk, high risk and unclear risk is made for the six items. The three items [ (1) (2) (5)] are used to evaluate the risk of bias of each included study, and the other three items are evaluated according to the different results of each included study, emphasizing that the different results in the same study are affected by bias to different extents. Two review authors independently applied the tool to each included study. Any discrepancies in judgments of risk of bias or justifications for judgements were resolved by discussion to reach consensus between the two review authors, with a third review author acting as an arbiter if necessary (Table 1). We used the GRADE approach (Grading of recommendations, development assessment and Evaluation) to rate certainty in the effect of MSCs on mortality, AEs and SAEs (Additional file 5: Table 5 and Additional file 6: Table 6).

### Effect measures

Since the trials data are dichotomous outcomes, we planned to analyze dichotomous outcomes by calculating the odds ratio (OR) of a successful outcome for each trial.

### Synthesis methods

We used the odds ratio to pool results to estimate the safety and efficacy of different kinds of stem cells in the treatment of ARDS and performed meta-analysis using Reman 5.3 software. The heterogeneity between studies was tested by Cochrane Q test and I^2^ test. If *P* > 0.1, when *I*^2^ ≤ 50%, it means that the homogeneity between studies is better, and then, the fixed-effects model is used; if *P* < 0.1, when *I*^2^ > 50%, it means that there is heterogeneity between the studies, and then, the random-effects model is used. If the number of included studies is more than 10, the publication bias analysis is performed by drawing a funnel chart, linear regression method, and rank correlation test. If the number of included studies is less than 10, then the publication bias is considered inevitable, and no correlation analysis is performed. Publication bias was considered inevitable because less than 10 trials were included in this study. Use sensitivity analysis to evaluate the stability of the research results. The results are statistically significant when *P* < 0.05.

## Results

**Fig. 1:**
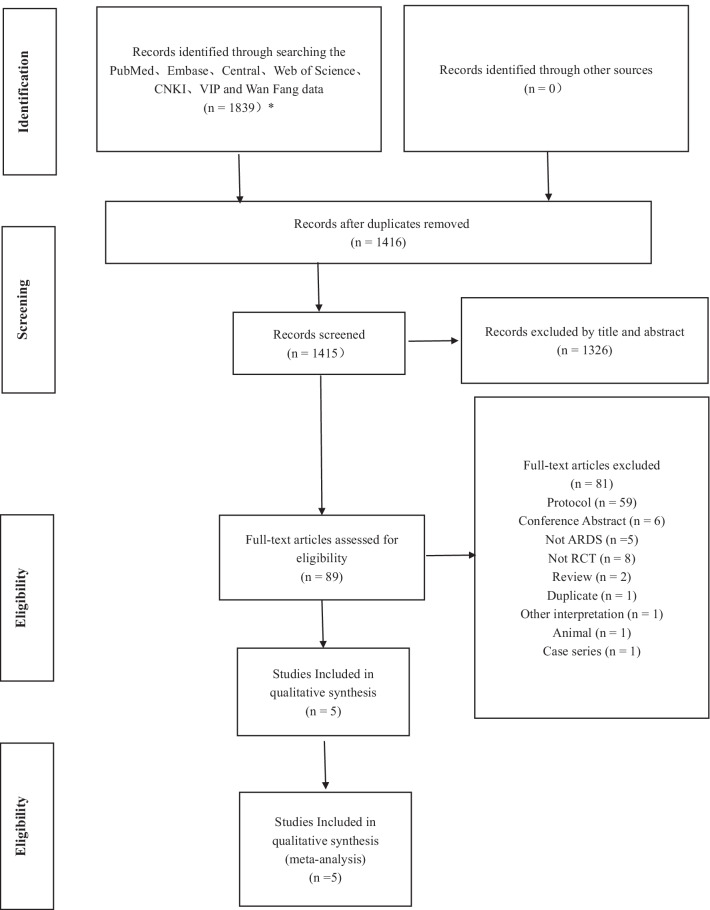
Flow chart of the selection process. Flow chart describing the selection steps of the systematic review and meta-analysis of comparing the safety, efficacy and biomarkers of mesenchymal stem cells in patients with ARDS, showing the number of studies excluded at each step, as well as the reasons for exclusion. *PubMed (*n* = 108), Embase (*n* = 381), Central (Cochrane library) (*n* = 176), Web of Science (*n* = 624), VIP (*n* = 132), Wanfang Data (*n* = 338), and China National Knowledge Infrastructure (*n* = 80). Ultimately, a total of 1839 were retrieved from the seven database. Of these 1839 studies, five articles were finally identified, including 5 quantitative studies

After searching the database, we found 1839 records. After removing duplicates, 1416 records were screened, of which 89 full texts were reviewed, and finally, 5 records were included [18–22]. Subsequently, we also searched the references of other current meta-analyses on MSCs studies. However, no additional articles meeting the inclusion criteria were found in these searches (Fig. 1).

Table 2 shows a summary of the patient, country, study type, age, percentage of males, PaO_2_/FiO_2_, SOFA score, primary ARDS Cause and lung injury score characteristics for each trial. Data from all included trials were obtained from published manuscripts. Also, Table 2 presents the specific details of the interventions and reports detailed data on mortality, subjects of adverse events or serious adverse events in each study.

**Table 2:** Characteristics of patients in included trials

Trail (Study ID)	Country	Study type	The number of patients, *n*			Age, year (mean/mean ± SD)		Male sex, *n*(%)
			MSC	Ctrl	Total	MSC	Ctr	
Monsel et al. [22]	France	Phase 2b RCT	21	24	45	64 ± 10.4	63.2 ± 11.4	37(82.2%)
Bellingan et al. [21]	UK	Phase 2a trial RCT	20	10	30	51 ± 14	59 ± 18	19 (63.3%)
Lanzoni et al. [20]	USA	Phase 1/2a trial RCT	12	12	24	58.6 ± 15.9	58.8 ± 11.6	13 (54.2%)
Matthay et al. [19]	USA	Phase 2a trial RCT	40	20	60	55 ± 17	55 ± 20	33 (55%)
Zheng et al. [18]	China	phase 1 trial RCT	6	6	12	66.7 ± 20.4	69.8 ± 9.1	11(91.7%)

Trail (Study ID)	PaO_2_/FiO_2_, mmHg (mean ± SD)/ median (IQR)		SOFA score (mean ± SD)		Primary ARDS Cause	Lung Injury Score (mean ± SD)	
	MSC	Ctrl	MSC	Ctrl		MSC	Ctrl
Monsel et al. [22]	156.2 ± 68.2	171.2 ± 72.9	5.5 ± 2.7	5.9 ± 2.7	COVID-19	3.0 ± 0.7	2.8 ± 0.5
Bellingan et al. [21]	173 ± 56.4	128 ± 35.1	10.9 ± 2.2^a^	12.2 ± 4.2^a^	Pneumonia	NR	NR
Lanzoni et al. [20]	124 (68–164)^b^	108.5 (68.5–165.5)^b^	NR	NR	COVID-19	NR	NR
Matthay et al. [19]	135.8 ± 32.3	143.3 ± 39	8.1 ± 3.3	6.9 ± 2.7	Pneumonia	3.1 ± 0.4	3.0 ± 0.5
Zheng et al. [18]	122.4 ± 42	103.5 ± 32.2	NR	NR	Pneumonia	NR	NR

^**a**^Modified SOFA, For SOFA scoring, all patients were assigned a nervous system domain score + 4 = Glasgow Coma Score (GCS) < 6

^**b**^PaO_2_/FiO_2_: median (IQR)

*RCT* Randomized Controlled Trial, *MSC* Mesenchymal stromal cell group, *Ctrl* Control group, *NR* not reported

**Fig. 2:**
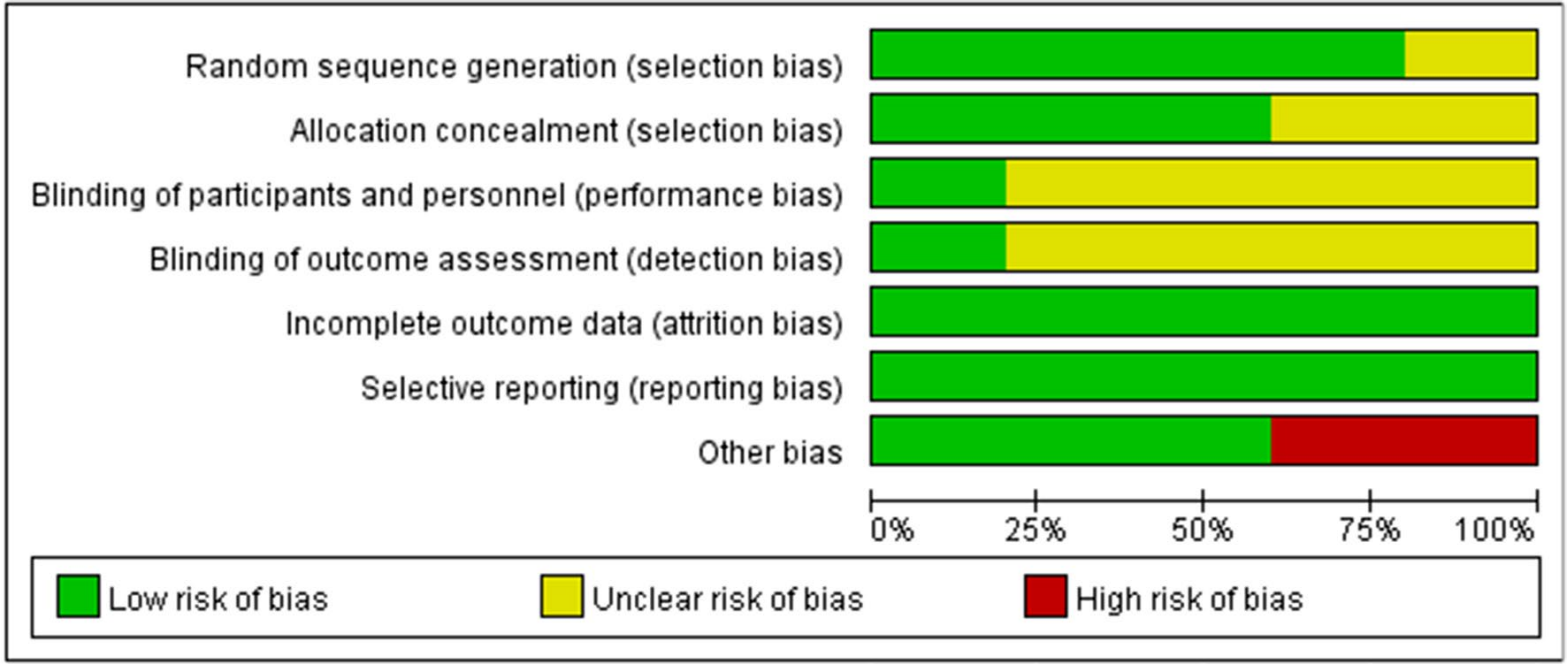
Risk of bias graph: review authors' judgments about each risk of bias item presented as percentages across all included studies

The risk of bias for each of included studies was assessed by the RoB2.0 tool (Fig. 2). Table 1 provides a summary of these assessments. In terms of overall risk of bias, the majority of studies (4/5) have different degrees of risk. We have also provided a detailed description of the justification for each assessment (Additional file 4: Table 4).

### Adverse events and Serious Adverse events

**Fig. 3:**
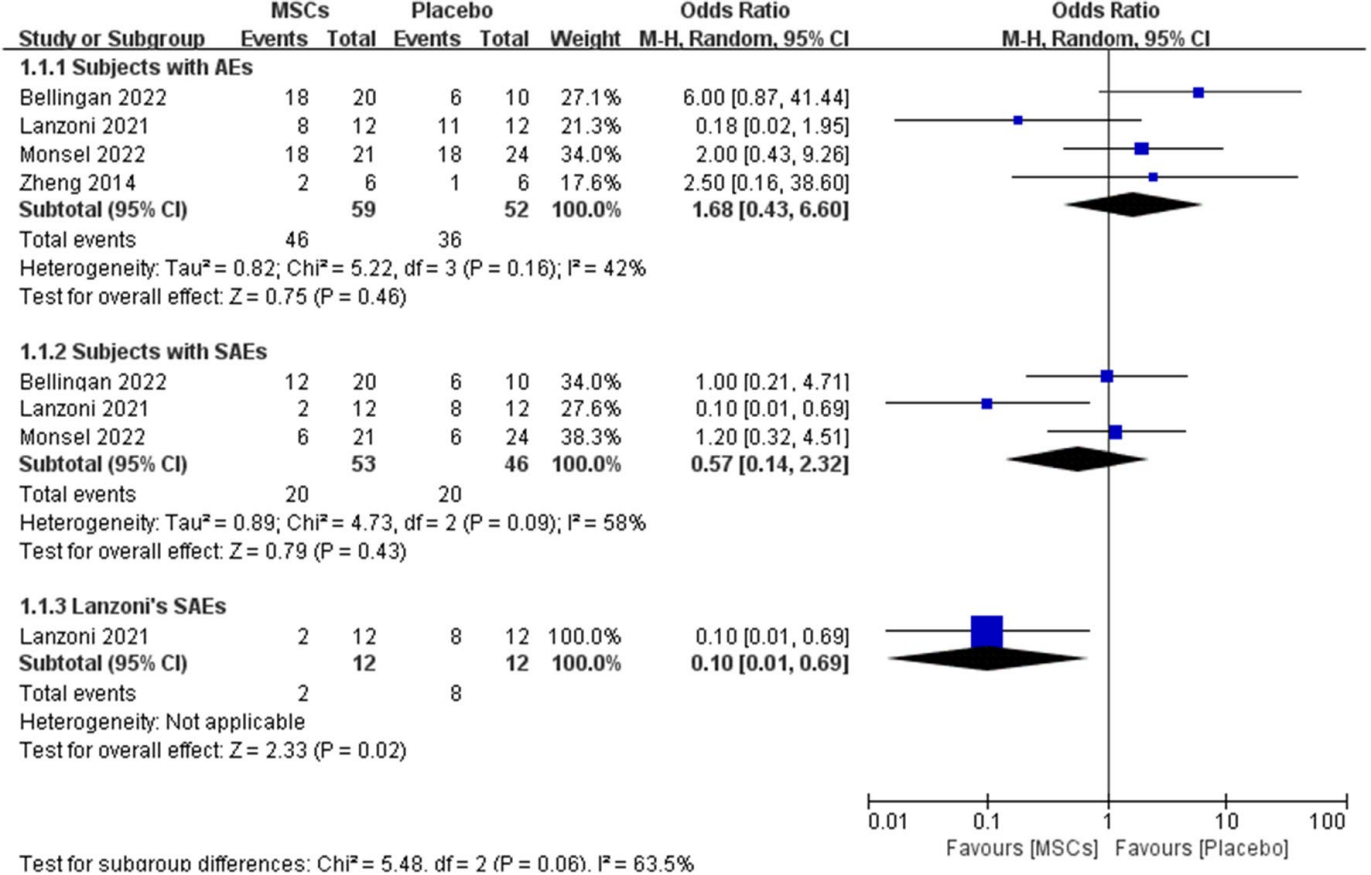
Forest plot of Subjects with AEs and SAEs. There was no statistical difference in the number of patients with AEs or SAEs after MSCs treatment compared with placebo. Three studies related to SAEs have some heterogeneity, and a random-effects model was used for statistical analysis. In the plane rectangular coordinate system, the forest plot takes a vertical invalid line (scale of abscissa is 0) as the center, describes the effect quantity and 95% CI of each study by using multiple line segments parallel to the horizontal axis, and describes the effect quantity and confidence interval of multiple studies by using a diamond. AEs, adverse events; SAEs, serious adverse events; MSCs, mesenchymal stem cells; CI, confidence interval

Four [18, 20–22] of the five included articles reported the occurrence of adverse events during follow-up and three [20–22] reported the occurrence of serious adverse events. A total of 99 individuals were randomly assigned to receive MSCs treatment, and 72 were randomly assigned to receive placebo treatment. One study [19] was excluded from analysis of adverse events due to lack of follow-up data on adverse events. In addition, two studies [18, 19] were excluded due to lack of follow-up data on serious adverse events. Of the 59 patients treated with MSCs, 46 experienced adverse events at follow-up, accounting for approximately 78% (46/59), of the 52 patients treated with placebo, 36 experienced adverse events at follow-up, accounting for approximately 69.2% (36/52), and there were three studies with OR > 1, accounting for approximately 75% (3/4) of the total studies, treatment with MSCs appeared to increase the occurrence of adverse events, but this result was not statistically significant (OR, 1.58; 95%CI, 0.64–3.91; *P* = 0.32), there was no significant heterogeneity in the adverse events across the 4 trials (χ2 = 5.22; *P* = 0.16; *I*^2^ = 42%) [18, 20–22]; of the 53 patients treated with MSCs, 20 experienced serious adverse events at follow-up, accounting for approximately 37.7% (20/53), of the 46 patients treated with placebo, 20 experienced serious adverse events at follow-up, accounting for approximately 55.6% (20/36), there were two studies [21, 22] with OR ≥ 1, accounting for about 66.7% (2/3) of the total studies, the study [20] with OR < 1 had certain statistical significance(OR, 0.10; 95%CI, 0.01–0.69; *P* = 0.02), but due to the small sample size, it did not indicate that MSCs treatment could reduce the occurrence of serious adverse reactions, in total, there appeared to be more serious adverse events with placebo treatment, but the results were not statistically significant (OR, 0.57; 95%CI, 0.14–2.32; *P* = 0.43), there was heterogeneity in the serious adverse events across the 3 trials (χ2 = 4.73; *P* = 0.09; *I*^2^ = 58%) [20–22] (Fig. 3).

### Mortality

**Fig. 4:**
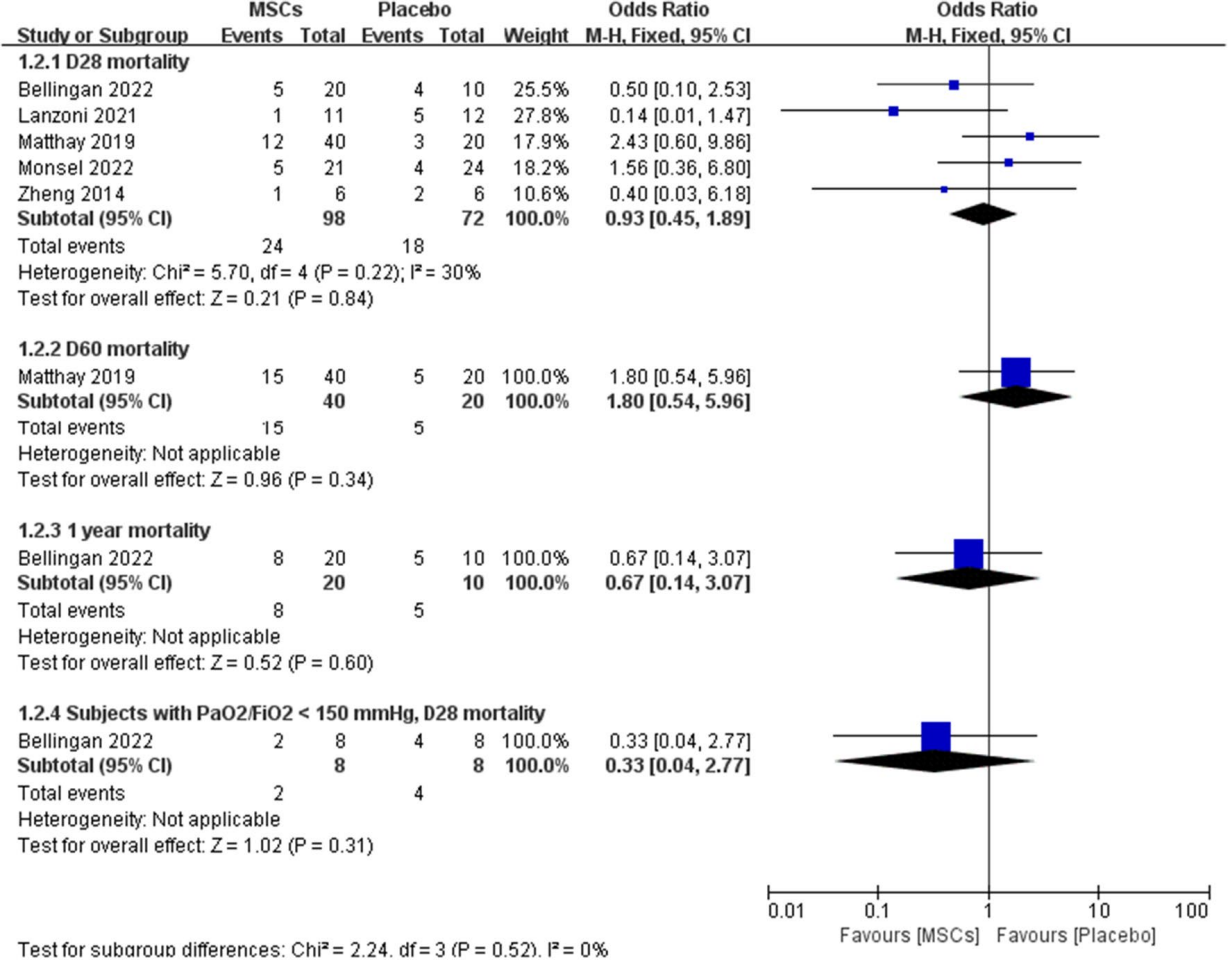
Forest plot of mortality. Mortality was not statistically significant between the two groups, either at short-term or long-term follow-up. No significant heterogeneity was observed in any of the five groups, and a fixed-effects model was used for statistical analysis. In the plane rectangular coordinate system, the forest plot takes a vertical invalid line (scale of abscissa is 0) as the center, describes the effect quantity and 95% CI of each study by using multiple line segments parallel to the horizontal axis, and describes the effect quantity and confidence interval of multiple studies by using a diamond. AEs, adverse events; SAEs, serious adverse events; MSCs, mesenchymal stem cells; CI, confidence interval

Five studies [18–22] reported 28-day all-cause mortality, one study [19] reported 60-day mortality, and one study [21] reported 1-year mortality. Two of the studies had an OR > 1 for 28-day mortality [19, 22], accounting for approximately 40% (2/5) of the total studies. 24 of the 98 patients in the MSCs group died, accounting for 24.5% (24/96), 18 of the 72 patients in the placebo group died, accounting for 25% (18/72). There seems to be no significant difference between the two groups in terms of 28 days mortality, but it lacks statistical significance (OR, 0.93; 95%CI, 0.45–1.89; *P* = 0.84), there was no significant heterogeneity in the mortality across the 5 trials (χ2 = 5.70; *P* = 0.22; *I*^2^ = 30%). Longer follow-up time (60 days) was reported in Matthay's study[19]. During the period from 28 to 60 days, the number of deaths increased by 3 [10.7% (3/28)] in MSCs group and 2 [11.8% (2/17)] in placebo group. Overall, the 60-day mortality was 37.5% (15/40) in the MSCs group and 25% (5/20) in the placebo group. Statistically, the two groups of data were not statistically significant (OR, 1.80; 95%CI, 0.54–5.96; *P* = 0.34). The study in Bellingan [21] was followed up for 1 year, and the follow-up result was that the mortality rate of the MSCs group [40% (8/20)] was lower than that of the placebo group [50% (5/10)], but its result was not statistically significant (OR, 0.67; 95%CI, 0.14–3.07; *P* = 0.60). Also, the study of Bellingan [21] gave the change of mortality in each group when PaO_2_/FiO_2_ was < 150 mmHg. Eight patients in the MSCs group met PaO_2_/FiO_2_ < 150 mmHg, and eight patients in the placebo group were eligible. There were 2 deaths [25% (2/8)] in the treatment group and 4 deaths [50% (4/8)] in the control group at follow-up 28. The mortality rate was lower in the critical treatment group, but this result was not statistically different possibly due to the small sample size (OR, 0.33; 95%CI, 0.04–2.77; *P* = 0.31) (Fig. 4). In summary, based on the current clinical study results, no statistical difference in mortality was observed between the treatment group and the control group whether short-term or long-term follow-up, which may be related to the small number of included studies, imbalance of baseline data in individual studies and only a few studies reporting long-term follow-up data.

### PaO_2_/FiO_2_

Four studies [18, 19, 21, 22] explained the changes in oxygenation index (PaO_2_/FiO_2_), all of which indicated that the increase in oxygenation index in the experimental group was higher than that in the control group, but also indicated that the difference between the two groups of data was not statistically significant. Refer to Additional file 3: Table 3 for the description of oxygenation index for each study.

### Biomarkers

All studies [18–22] reported on plasma biomarkers in patients treated with MSCs versus placebo in detail. First, Zheng's study [18] in 2014 pointed out that SP-D, IL-6 or IL-8 levels were similar between day 0 and day 5 in the placebo group, while the inflammatory factors levels (SP-D, IL-6 or IL-8) in the MSCs group were significantly decreased compared with the baseline data on day 0, and the decrease in SP-D was statistically significant. However, the study [18] also showed that the changes in inflammatory factors were not statistically different between the two groups. Then, Matthay's study [19] in 2019, at 6 h after the start of infusion, the decrease in angiopoietin 2 concentration in plasma was significantly greater in the MSC group than in the placebo group (*P* = 0.005). Recently, Lanzoni's studies [20] observed only the UC-MSC-treated group showed a consistent decrease in inflammatory markers. At day 6, a significant difference in the concentration of GM-CSF, IFNg, IL-5, IL-6, IL-7, TNFa, TNFb, PDGF-BB, and RANTES was observed in a comparison between groups. Bellingan's study [21] also demonstrated an average decrease in inflammatory biomarkers (IFN-gamma, IL-1 beta, IL-1R2, IL 6, IL 12, KGF, PD-1, RAGE, and TNF-alpha) in the cells group on day 7, but Bellingan's study did not statistically analyze the changes. Monsel's study [22] provides a volcano plot of plasma biomarkers from 0 to 14 days, as can be seen from the plot, by the 14th day, IL-7, IL-10, IP-10, IL-18, RAGE and MCP-2 were decreased in all groups, while IL-9, IL-10 and IL-17F were decreased in the cell group and statistically different from the control group. (Additional file 3: Table 3).

## Discussion

In this study, we analyzed the associations between MSCs and ARDS using a meta-analysis to obtain a powerful conclusion, and we summarized the latest RCT clinical studies on MSCs in ARDS on the basis of previous studies and performed a detailed analysis of their biomarkers to update the previous meta-analysis. Although Qu has done a similar meta-analysis on the safety and efficacy of MSCs in the treatment of COVID-19-associated ARDS in 2020, Qu's study included few RCTs and lacked a detailed analysis of biomarkers [17].

Meta-analysis showed that MSCs treatment reduced the occurrence of serious adverse events in ARDS patients compared with placebo, but was accompanied by an increased risk of adverse events (SAEs, OR < 1; AEs, OR > 1), although this result was not statistically different (SAEs, *P* = 0.32; AEs, *P* = 0.43), and all five included studies stated that the occurrence of adverse events and serious adverse events in the treatment group was mainly related to the patient's current disease, without significant association with MSCs [18–22]. In Monsel's study [22], only one of the adverse events (diarrhea) was considered related to the treatment process of MSCs; in Belligan's study [21], the baseline data of the two groups were not completely balanced, the modified SOFA score of the cell group was lower, the oxygenation index was higher, and these two were statistically different from the placebo group, and there was one adverse event (grade 1 fever) considered related to MSCs treatment in Belligan's study; in Lanzoni's study [20], the baseline data of the two groups were basically balanced, only one adverse event was considered related to infusion within 6 h in the cell group and two in the placebo group, and it was considered that MSCs treatment could reduce the occurrence of SAEs in the cell group; in Zheng's study [18], only two adverse events (diarrhea, rash) were considered related to MSCs treatment in the cell group, and these two adverse events resolved spontaneously at 48 h and 24 h, respectively. In general, although the incidence of adverse events in the cell group was considered to be higher in the included studies, but it was considered to be unrelated to the treatment with MSCs after respective analysis, a few related adverse events were mild in severity, had a lower incidence, and had a tendency of self-healing; for serious adverse events, the study suggested that the treatment with MSCs had the potential efficacy of reducing the occurrence of serious adverse events, but this needs to be corroborated by standard randomized controlled trials with larger sample size and follow-up time. At present, it is believed that the safety of MSCs in the treatment of ARDS is fair and basically consistent with the conclusions of previous studies [23–26], and these studies all acknowledged the safety of MSCs in the treatment of ARDS.

As of April 2, 2022, a total of 476 million COVID-19 cases have been diagnosed worldwide, with a cumulative number of 6.12 million deaths reported and a mortality rate of approximately 1.3%. [Data were obtained from the National Health Commission of China, the World Health Organization, official epidemic notification and authoritative media reports from various countries (regions), it is summarized by the official media of China and published on the epidemic map platform.] With the worldwide pandemic of COVID-19, the number of patients with ARDS has increaed dramatically [27]. According to an epidemiological study from 50 countries in 2016, the prevalence of ARDS in ICU is 10.4%, hospital mortality was 34.9% for those with mild, 40.3% for those with moderate, and 46.1% for those with severe ARDS [28]. Arguably, in the context of the current COVID-19 pandemic, reducing in-hospital mortality in ARDS is a great challenge and urgent need today. The result of this study indicates the potential efficacy of MSCs therapy to reduce in-hospital mortality in moderate and severe ARDS, although there is no statistical support for this result. And, most of the included studies (3/5) considered the cell group to have a lower mortality rate [18, 20, 21]. Although Matthay's study [19] results concluded that the mortality rate of the cell group was higher than that of the placebo group [MSCs group, 30% (12/40); placebo group, 15% (3/20)], this result may be related to the more severe disease in the cell group, which can be confirmed by the lower oxygenation index (MSCs group, 135.8 ± 32.3; placebo group, 143.3 ± 39) and higher SOFA score (MSCs group, 8.1 ± 3.3; placebo group, 6.9 ± 2.7) in the cell group than in the placebo group. In another study [22], which stated that the mortality rate of the cell group was higher than that of the placebo group, the authors stated that there was no statistical difference in mortality between the two groups. And the topic of Monsel's study is COVID-19-related ARDS, which is not completely consistent with the disease development process of ARDS in the traditional sense, such as the prevalence of coagulopathy and venous thrombosis in COVID-19-related ARDS [29–32]. In severe cases of ARDS, Bellingan's study [21] found that when PaO_2_/FiO_2_ was < 150 mmHg, the mortality rate was significantly lower after MSCs treatment, and although it was not statistically different because of the small sample size, the potential therapeutic effect of MSCs was undeniable. It has also been shown in several large animal trials and clinical trials that MSCs therapy can reduce the mortality of ARDS [33–35]. The results of this study suggest that MSCs therapy has the possibility of reducing the mortality of ARDS, which is consistent with previous animal experiments and clinical trials. However, it is also recommended to conduct a clinical trial with a large sample size to fully confirm this efficacy.

Oxygenation index, as an important indicator of respiratory function and diagnostic criteria for ARDS, was reported and analyzed as a secondary outcome measure in four of the five included studies [18, 19, 21, 22]. The study showed that the oxygenation index was improved after treatment in both the cell group and the placebo group, and there was no statistical difference between the two groups of data. Since both groups of patients received mechanical ventilation, and the improvement of oxygenation index by mechanical ventilation has been confirmed by most studies [3, 4], it is difficult to say from the current research evidence that MSCs have a clear therapeutic effect of improving oxygenation index. And whether oxygenation index can play a role in judging the prognostic indicators is still somewhat controversial [36], mainly because oxygenation index is highly susceptible to limitations such as atmospheric pressure and FiO_2_ and so on [37]. Monsel's study [22] suggested that MSCs have the ability to promote lung tissue repair and improve oxygenation, the lack of significant difference in the change of oxygenation index between the two groups was interpreted as the severity of lung injury exceeded the repair effect of MSCs when the patient's respiratory failure was severe enough to require invasive mechanical ventilation or ventilator support. According to the pooled results of this study, there are many intervention factors for oxygenation index. Although it has been shown from animal tests that MSCs have the effect of improving lung injury, which can indirectly predict that MSCs may improve oxygenation by improving lung injury, and there is no direct evidence that MSCs have the effect of improving oxygenation.

The results vary widely between studies on biomarkers, and there is no uniform standard for the types of biomarkers observed. The most important reason for the difference may be that the biomarkers were from plasma rather than from the lungs. A recent study has shown that the use of nonbronchoscopic bronchoalveolar lavage as airspace biomarker may represent a lung-specific therapeutic effect than plasma biomarkers [38]. The five studies [18–22] had some contradictions to the reporting of biomarkers, for example, Zheng's study [18] indicated that SP-D decreased in the cell group and was different from the placebo group, while Belligan's study [21] found that SP-D increased in the cell group but decreased in the placebo group. In general, plasma biomarkers such as IL-6, IL-8, RAGE, and angiopoietin-2 (Ang-2) decreased more significantly in cells groups agreed with most of the included studies. Ang-2, on the other hand, is recognized as a mediator and biomarker of pulmonary and systemic vascular injury. And the concentration of Ang-2 has important predictive value for the development of ARDS [39–41]. Wick designed a randomized controlled study on airspace biomarkers and plasma biomarkers to determine whether airspace biomarkers can increase the value of plasma biomarkers and whether airspace biomarkers provide mechanistic evidence for MSCs in the treatment of ARDS [38]. Wick’ study has shown that the concentrations of airspace biomarkers were significantly different from those in plasma. The concentrations of IL-8, IL-6, and RAGE were significantly lower in plasma than in airspaces, while the concentrations of sTNFR-1 and Ang-2 were significantly lower in airspaces than in plasma [38]. Both Airspace Ang-2 and airspace RAGE were positively correlated with airspace total protein [38]; in experimental models of MSC therapy for ARDS and clinical studies of ARDS, the concentration of total protein in the airspaces is a good biomarker of lung endothelial cell and epithelial protein permeability [42–46]. Compared to plasma Ang-2 and plasma RAGE, higher airspace Ang-2 is associated with fewer ventilator-free days (VFDs), while higher airspace RAGE is associated with higher radiographic assessment of lung oedema (RALE) score [38]. There was no significant statistical difference in plasma biomarkers between the treatment placebo group, which may be related to the fact that plasma biomarkers are not only derived from lung tissues, but also from other tissues [47]. Therefore, for some biomarkers, airspace biomarkers may reflect different biological processes compared to plasma biomarkers [38]. Plasma IL-8 is an important biomarker for the assessment of ARDS [48], but IL-8 in plasma does not accurately reflect the inflammatory environment in the lungs. Similarly, there is a lack of correlation between airspace and plasma Ang-2 concentrations [49]. At 48 h after treatment, the values of airspace biomarkers in the MSCs group were very low, and most of them were statistically different compared with the placebo group, in which airspace Ang-2 levels were significantly reduced (*P* = 0.0076), while plasma biomarkers were not different from the placebo group [38]. So, plasma biomarkers may reflect the overall level of the disease, and airspace biomarkers represent lung-specific therapeutic effects [38]. Although based on the current study, it could not be fully demonstrated that the plasma biomarkers in the MSCs group were significantly different from those in the placebo group after treatment, in terms of airspace biomarkers, it has been proposed that MSCs treatment can significantly reduce airspace biomarkers within 48 h. Among them, airspace Ang-2, airspace RAGE have some specificity in assessing the therapeutic effect of lung injury. In future biomarker studies, more attention to airspace biomarkers may be a better option.

In this study, based on the latest clinical randomized controlled study, the safety, efficacy and biomarkers of MSCs in the treatment of ARDS were comprehensively and comprehensively analyzed, providing a more comprehensive theoretical basis for future studies. This study also has some limitations. First, only five studies were included, although all five studies were high-quality randomized controlled studies; second, the number of subjects included in each study was small, resulting in that the total number of subjects included in the meta-analysis was only 171, which made the results of this study possibly inconsistent with the results of future studies; finally, two of the included studies indicated the use of steroids during treatment [21, 22], which have a similar effect to MSCs and have a cytotoxic effect on MSCs, which may have some impact on the efficacy of MSCs, but in some studies, it was pointed out that steroids (e.g., dexamethasone) have a small effect on the activity of cells [50], and in one study, MSCs have been used as rescue therapy for acute graft-versus-host disease with severe steroid resistance [51]. So although there is steroid use, this does not deny the therapeutic effect of MSCs.

## Conclusion

Compared with the placebo group, MSCs have considerable safety in the treatment of ARDS and have the potential to reduce the mortality of moderate and severe ARDS. Airspace biomarkers represent lung-specific treatment efficacy than plasma biomarkers, and airspace Ang-2 and airspace RAGE have some specificity in assessing the therapeutic effect of ARDS. More and larger studies are also necessary to further confirm the safety and efficacy of MSCs and the predictive value of airspace biomarkers.

## Supplementary Information

**Additional file 1**. Search strategy.

**Additional file 2**. Data characteristics in Included Trials.

**Additional file 3**. PaO_2_/FiO_2_ and Biomarkers characteristics in Included Trials.

**Additional file 4**. Characteristics of included studies.

**Additional file 5**. Quality of GRADE.

**Additional file 6.** Summary of Findings.

## Data Availability

Template data collection forms, data extracted from included studies and data used for all analyses have been included in the Supplementary Information.

## Abbreviations

MSCs: Mesenchymal stromal cells
ARDS: Acute respiratory distress syndrome
RCT: Randomized controlled trial
AEs: Adverse events
SAEs: Serious adverse events
OR: Odds ratio
CI: Confidence interval
MSC MVs: Mesenchymal stem cell microvesicles
EMT: Epithelial–mesenchymal transition
COVID-19: Coronavirus disease 2019
SOFA score: Sequential organ failure assessment score
ALI: Acute lung injury
GRADE: Grading of recommendations, development assessment and Evaluation
APRV: Airway pressure release ventilation
AMs: Alveolar macrophages
ECMO: Extracorporeal membrane oxygenation
HGF: Hepatocyte growth factor
DCs: Dendritic cells
