# Supplementary material for: Safety, efficacy and biomarkers analysis of mesenchymal stromal cells therapy in ARDS: a systematic review and meta-analysis based on phase I and II RCTs
Source: Stem Cell Res Ther. 2022 Jun 25;13:275. doi: 10.1186/s13287-022-02956-3 (PMC9233855; doi:10.1186/s13287-022-02956-3)
Supplement: Supplementary file 1 — Additional file 1. Search strategy. [file 13287_2022_2956_MOESM1_ESM.docx]

Pubmed

1 ARDS [tiab]

2 Acute Respiratory Distress Syndrome [tiab]

3 ALI [tiab]

4 acute lung injury [tiab]

5 Shock Lung [tiab]

6 Respiratory Distress Syndrome [tiab]

7 1 or 2 or 3 or 4 or 5 or 6

8 mesenchymal stromal cell [tiab]

9 stromal cell [tiab]

10 mesenchymal stem cell [tiab]

11 stem cell [tiab]

12 Mesenchymal Progenitor Cell [tiab]

13 Progenitor Cell [tiab]

14 MSC [tiab]

15 MSCs [tiab]

16 8 or 9 or #0 or 11 or 12 or 13 or 14 or 15

17 randomized controlled trial [pt]

18 controlled clinical trial [pt]

19 randomized [tiab]

20 placebo [tiab]

21 randomly [tiab]

22 trial [tiab]

23 groups [tiab]

24 17 or 18 or 19 or 20 or 21 or 22 or 23

25 animals[mh] not（humans[mh] and animals[mh])

26 7 and 16 and 24

27 26 not 25

Embase

#1 Acute Respiratory Distress Syndrome/

#2 ARDS.ti,ab.

#3 Acute Respiratory Distress Syndrome.ti,ab.

#4 ALI.ti,ab.

#5 acute lung injury.ti,ab.

#6 Shock Lung.ti,ab.

#7 Respiratory Distress Syndrome.ti,ab.

#8 or/#1-#7

#9 mesenchymal stromal cell/

#10 mesenchymal stromal cell.ti,ab.

#11 stromal cell.ti,ab.

#12 mesenchymal stem cell.ti,ab.

#13 stem cell.ti,ab.

#14 Mesenchymal Progenitor Cell.ti,ab.

#15 Progenitor Cell.ti,ab.

#16 MSC.ti,ab.

#17 MSCs.ti,ab.

#18 or/#9-#17

#19 randomized controlled trial/

#20 controlled clinical trial/

#21 double blind procedure/

#22 single blind procedure/

#23 randomized.ti,ab.

#24 placebo.ti,ab.

#25 randomly.ti,ab.

#26 trial.ti,ab.

#27 groups.ti,ab.

#28 or/#19-#26

#29 animal/ not (humans/ and animal/)

#30 #8 and #18 and #28

#31 #29 not #28

CENTRAL (Cochrane Library) search strategy

#1 MeSH descriptor: [Respiratory Distress Syndrome] explode all trees

#2 ARDS:ti,ab,kw

#3 Acute Respiratory Distress Syndrome:ti,ab,kw

#4 ALI:ti,ab,kw

#5 acute lung injury:ti,ab,kw

#6 Shock Lung:ti,ab,kw

#7 Respiratory Distress Syndrome:ti,ab,kw

#8 (#1 or #2 or #3 or #4 or #5 or #6 or #7)

#9 MeSH descriptor: [Mesenchymal Stem Cells] explode all trees

#10 mesenchymal stromal cell:ti,ab,kw

#11 stromal cell:ti,ab,kw

#12 mesenchymal stem cell:ti,ab,kw

#13 stem cell:ti,ab,kw

#14 Mesenchymal Progenitor Cell:ti,ab,kw

#15 Progenitor Cell:ti,ab,kw

#16 MSC:ti,ab,kw

#17 MSCs:ti,ab,kw

#18 (#9 or #10 or #11 or #12 or #13 or #14 or #15 or #16 or #17)

#19 #8 and #18

Web of science

#1 TS=(mesenchymal stromal cell) OR TS=( stromal cel) OR TS=(mesenchymal stem cell ) OR TS=(stem cell) OR TS=(Mesenchymal Progenitor Cell ) OR TS=(Progenitor Cell) OR TS=(MSC) OR TS=( MSCs)

#2 TS=(ARDS) OR TS=(Acute Respiratory Distress Syndrome) OR TS=(ALI) OR TS=(acute lung injury) OR TS=(Shock Lung) OR TS=(Respiratory Distress Syndrome)

#3 TS=(randomized ) OR TS=(placebo) OR TS=(randomly) OR TS=(trial) OR TS=(groups)

#4 #1 and #2 and #3

#5 TS=(human) AND TS=(animal)

#6 TS=(animal)

#7 #5 not #4

#8 #4 not #7

From the beginning of literature inclusion to March 17, 2022
