# Supplementary material for: Safety, efficacy and biomarkers analysis of mesenchymal stromal cells therapy in ARDS: a systematic review and meta-analysis based on phase I and II RCTs
Source: Stem Cell Res Ther. 2022 Jun 25;13:275. doi: 10.1186/s13287-022-02956-3 (PMC9233855; doi:10.1186/s13287-022-02956-3)
Supplement: Supplementary file 2 — Additional file 2. Data characteristics in Included Trials. [file 13287_2022_2956_MOESM2_ESM.docx]

| **Supplementary Table 2 Data characteristics in Included Trials** | | | | | | | | | |
| --- | --- | --- | --- | --- | --- | --- | --- | --- | --- |
| Trials | MSCs source | Administration route | DOSE | Tevent | Tnoevent | Ttotal | Cevent | Cnoevent | Ctotal |
| D28 mortality | | | | | | | | | |
| Monsel 2022 | Umbilical cord | intravenous infusion | 1 x 10^6^MSCs/kg | 5 | 16 | 21 | 4 | 20 | 24 |
| Bellingan 2022 | Bone marrow | intravenous infusion | 9 × 10^8^MAPCs | 5 | 15 | 20 | 4 | 6 | 10 |
| Lanzoni 2021 | Umbilical cord | intravenous infusion | 100 ± 20 × 10^6^ MSCs | 1 | 10 | 11**^a^** | 5 | 7 | 12 |
| Matthay 2019 | Bone marrow | intravenous infusion | 10 × 10^6^ MSCs/kg | 12 | 28 | 40 | 3 | 17 | 20 |
| Zheng 2014 | Adipose | intravenous infusion | 1 × 10^6^ MSCs/kg | 1 | 5 | 6 | 2 | 4 | 6 |
| Subjects with Adverse events（AEs） | | | | | | | | | |
| Monsel 2022 | umbilical cord | intravenous infusion | 1 x 10^6^MSCs/kg | 18 | 3 | 21 | 18 | 6 | 24 |
| Bellingan 2022 | Bone marrow | intravenous infusion | 9 × 10^8^MAPCs | 18 | 2 | 20 | 6 | 4 | 10 |
| Lanzoni 2021 | Umbilical cord | intravenous infusion | 100 ± 20 × 10^6^ MSCs | 8 | 4 | 12 | 11 | 1 | 12 |
| Matthay 2019 | Bone marrow | intravenous infusion | 10 × 10^6^ MSCs/kg | NA | NA | NA | NA | NA | NA |
| Zheng 2014 | Adipose | intravenous infusion | 1 × 10^6^ MSCs/kg | 2 | 4 | 6 | 1 | 5 | 6 |
| Subjects with Serious Adverse events（SAEs） | | | | | | | | | |
| Monsel 2022 | umbilical cord | intravenous infusion | 1 x 10^6^MSCs/kg | 6 | 15 | 21 | 6 | 18 | 24 |
| Bellingan 2022 | Bone marrow | intravenous infusion | 9 × 10^8^MAPCs | 12 | 8 | 20 | 6 | 4 | 10 |
| Lanzoni 2021 | Umbilical cord | intravenous infusion | 100 ± 20 × 10^6^ MSCs | 2 | 10 | 12 | 8 | 4 | 12 |
| 1 year mortality | | | | | | | | | |
| Bellingan 2022 | Bone marrow | intravenous infusion | 9 × 10^8^MAPCs | 8 | 12 | 20 | 5 | 5 | 10 |
| D60 mortality | | | | | | | | | |
| Matthay 2019 | Bone marrow | intravenous infusion | 10 × 10^6^ MSCs/kg | 15 | 25 | 40 | 5 | 15 | 20 |
| Subjects with PaO2/FiO2 < 150 mmHg D28 mortality | | | | | | | | | |
| Bellingan 2022 | Bone marrow | intravenous infusion | 9 × 10^8^MAPCs | 2 | 6 | 8 | 4 | 4 | 8 |

**^a^** One subject (Subject #11) in the UC-MSC treatment group died as a result of a failed endotracheal intubation; NA: not applicable; NR: not reported

Tevent: Subject of Treatment event; Tnoevent: Subject of Treatment no event; Cevent: Subject of Control event; Cnoevent: Subject of Control no event;
