# Supplementary material for: Safety, efficacy and biomarkers analysis of mesenchymal stromal cells therapy in ARDS: a systematic review and meta-analysis based on phase I and II RCTs
Source: Stem Cell Res Ther. 2022 Jun 25;13:275. doi: 10.1186/s13287-022-02956-3 (PMC9233855; doi:10.1186/s13287-022-02956-3)
Supplement: Supplementary file 3 — Additional file 3. PaO2/FiO2 and Biomarkers characteristics in Included Trials. [file 13287_2022_2956_MOESM3_ESM.docx]

| **Supplementary Table 3 PaO_2_/FiO_2_ and Biomarkers characteristics in Included Trials** | | |
| --- | --- | --- |
| Trails | PaO_2_/FiO_2_ | Biomarkers |
| Monsel 2022 | PaO2/FiO2 ratios increased significantly from D0 to D7 in the UC‑MSC group (respectively, 156.2 ± 68.2 vs 188.3 ± 74.2; Wilcoxon signed‑rank exact test). The placebo group’s PaO2/FiO2 ratios on D0 and D7 were comparable (respectively, 171.2 ± 72.9 vs 169.8 ± 85.6, Wilcoxon signed‑rank exact test). | On the 14th day, IL-7, IL-10, IP-10, IL-18, RAGE and MCP-2 were decreased in all groups, while IL-9, IL-10 and IL-17F were decreased in the cell group and statistically different from the control group. |
| Bellingan 2022 | Increases in PaO2/FiO2 were seen in both groups, indicating improving lung function among ventilated survivors. | Several pro-inflammatory biomarkers (IFN-gamma, IL-1 beta, IL-1R2, IL 6, IL 12, KGF, PD-1, RAGE, and TNF-alpha) decreased on average through Day 7 in the cell treatment group and increased through Day 7 among the placebo group. SP-D and TSP-1 that increased through Day 7 in the cell treatment group. |
| Lanzoni 2021 | None | We observed that a consistent decrease in inflammatory markers only in the UC-MSC treatment group. In a comparison between groups at day 6, we observed significant differences in the concentration of GM-CSF, IFNg, IL-5, IL-6, IL-7, TNFa, TNFb, PDGF-BB, and RANTES (P <0 .05). In the longitudinal analysis, inflammatory cytokine concentrations showed marked and statistically significant decreases from day 0 to day 6 only in the UC-MSC treatment group |
| Matthay 2019 | Oxygenation index seemed to improve from baseline to 2 days after the start of infusion in the intermediate and highest tertiles of MSC viability, albeit non-significantly. | The decrease of concentrations of angiopoietin 2 in plasma was significantly greater in the MSC group than in the placebo group 6h after the start of infusion (P=0.005). No changes from baseline were seen for IL-6, IL-8, RAGE, or protein C concentrations at 6 or 24h in either group. |
| Zheng 2014 | Significant improvements in oxygenation index from baseline were observed in all data points in the MSCs group. In the placebo group, there were no significant improvements at days 5 (p = 0.05) and 7 (p = 0.05) as compared to baseline. The PaO2/FiO2 did not differ significantly between MSCs and placebo groups at all time points. | In the placebo group, SP-D, IL-6 or IL-8 levels were similar between day 0 and day 5. In the MSCs group there was a decrease in the biomarkers (SP-D, IL-6 and IL-8), but only SP-D was statistically significant (P=0.027). |
