# Supplementary material for: Safety, efficacy and biomarkers analysis of mesenchymal stromal cells therapy in ARDS: a systematic review and meta-analysis based on phase I and II RCTs
Source: Stem Cell Res Ther. 2022 Jun 25;13:275. doi: 10.1186/s13287-022-02956-3 (PMC9233855; doi:10.1186/s13287-022-02956-3)
Supplement: Supplementary file 4 — Additional file 4. Characteristics of included studies. [file 13287_2022_2956_MOESM4_ESM.docx]

| Supplementary Table 4 Characteristics of included studies | | | | |
| --- | --- | --- | --- | --- |
| ***Monsel 2022*** | | | | |
| Methods | Prospective, Phase 2b, multicentre, double‑blind, randomized, placebo‑controlled trial  The study was conducted in ten ICUs in eight French university hospitals  Period of Inclusion: April 6, 2020, to October 29, 2020 | | | |
| Participants | **Inclusion criteria**  Age > 18 years  Reverse transcriptase–polymerase chain reaction-confirmed SARS–CoV‑2 infection  Berlin criteria‑defined acute respiratory distress syndrome for < 96h Respiratory support (invasive or non‑invasive mechanical ventilation, and/or high‑flow nasal oxygenation) with positive end  ‑expiratory pressure equivalent ≥ 5 cm H2O  **Exclusion criteria**  Age < 18 years  Acute respiratory distress syndrome present for > 96h Pulmonary fibrosis  Pulmonary hypertension (WHO classification class III or IV)  Pulmonary embolism within the previous 3 months  Extracorporeal membrane oxygenation or life support  Immunocompromised status including use of immunosuppressive medications  Pregnancy or breastfeeding  Treatment for cancer in the past 2 years  Underlying medical condition with life expectancy < 6 months  Moderate‑to‑severe liver disease (Child–Pugh score > 12)  Severe chronic lung disease with the use of home oxygen and/or partial arterial pressure of carbon dioxide > 50 mm Hg  Patients not committed to full support (i.e., had do not resuscitate or limit life support orders)  Participation in another trial of COVID‑19 therapeutics  **Baseline characteristics**  N = 45, mean age of 63.6 years and 82.2% of men | | | |
| Interventions | Each patient received three intravenous infusions of 10^6^ UC-MSCs/kg (maximum dose set at 80 × 10^6^ cells per infusion) or placebo on D1, D3 ± 1 and D5 ± 1. All patients were monitored for any changes of pre-specified  respiratory or cardiovascular parameters and were ventilated according to the modified ARDS Network lower tidal volume protocol. Management of ARDS, septic shock and other organ failures followed international guidelines. | | | |
| Outcomes | **Primary endpoint**  Respiratory improvement assessed as the partial pressure of oxygen to fractional inspired oxygen (PaO2/FiO2)-ratio change between baseline (D0) and D7 post-randomization.  **Secondary endpoints**  All-cause mortality at D28  Number of ventilator-free days to D28  Duration of ventilation in patients alive on D28  Number of intensive care-free days to D28  Number of organ-failure free days to D28  SOFA score  Lung injury score  Driving pressure  Respiratory lung compliance  **Safety endpoints**  Number of adverse events attributable to UC-MSC administration,  The number of pre-specified infusion-related adverse events (listed below) during the infusion of study treatments (ATMP or placebo)  The 6 hours following infusion onset, during the 24 hours following each administration on D1, D3 ( 1 day) and D5 ( 1 day), during the treatment period from D1 to D7, and any adverse events associated with UC-MSC infusion from D1 to D28  *During the infusion of study treatments (ATMP or placebo)*:  A clinical picture compatible with a transfusion incompatibility or an infusion-linked event (urticaria, bronchospasm…)  *During the 6 hours following infusion onset of study treatments*:  Onset of hemodynamic instability requiring administration of noradrenaline ≥2 mg/h; decreased blood pressure necessitating a higher dose of noradrenaline ≥2 mg/h compared to its initial value  Ventricular tachycardia, ventricular fibrillation or cardiac arrest  Development of severe hypoxia (partial pressure of oxygen to fractional inspired oxygen (PaO2/FiO2)) ≤60 mm Hg or ≥50% of its preinfusion value.  *During the 24 hours following each infusion :*  Any cardiac arrest.  *During the treatment period from day (D) 1 to D7*:  Massive pulmonary embolism with hemodynamic repercussions  Deterioration of arterial oxygenation with severe hypoxia unexplained by the evolution of the SARS-CoV-2-induced ARDS and requiring implantation of venovenous-extracorporeal membrane oxygenation  Any clinical evidence identified by the physician as unusual by its characteristics, frequency, or severity compared to the clinical evolution of ARDS. | | | |
| ***Cochrane Risk of bias*** | | | | |
| **Bias** | | | **Assessment** | **Support** |
| 1. Random sequence generation (selection bias) | | | Low risk | **Quote:** “a protocol amendment to 1:1. Randomization was stratified according to age (≤ 70 vs > 70 years) and the inclusion-day (D0) Sequential Organ-Failure Assessment (SOFA) score (≤ 11 vs > 11). Randomization were using a web-based electronic case-report form (Cleanweb Telemedicine Technologies™) on a sponsor-operated secure server”  **Comment:**  The method used to guarantee randomization seems to be adequate. |
| 1. Allocation concealment (selection bias) | | | Low risk | **Quote:** “Labelling and distributions of UC-MSCs and placebo pouches were rigorously identical, with both being pre-conditioned in opaque bags.”  **Comment:** The method is adequate. |
| 1. Blinding of participants and personnel   (performance bias) | | | Low risk | **Quote:** “To maintain blinding of the investigators and clinicians, conditioning, labelling and distributions of UC-MSCs and placebo pouches were rigorously identical, with both being pre-conditioned in opaque bags. The intravenous infusion tubing set was not blinded per se. Only the bag was covered. However, it has been considered that, given the dilution of the cell suspension, and, thus, the transparent appearance of the fluid, it was not possible for the treating staff to ascertain whether or not the patient received the treatment or the placebo only by looking at the infusion set.”  **Comment:** The method is adequate. |
| 1. Blinding of outcome assessment (detection bias) | | | Low risk | **Quote:** “Investigators remained unaware of treatment allocations until the database was fully cleansed and exported for statistical analysis.”  **Comment:**  The method seems to be adequate. |
| 1. Incomplete outcome data (attrition bias) | | | Low risk | **Quote:** “UC-MSCs: 1 Withdrew consent.”  **Comment:** One of the experimental group dropped out, which had little effect on the results |
| 1. Selective reporting (reporting bias) | | | Low risk | **Comment:** in accordance with  the protocol available in clinical trials.gov. (NCT04333368) |
| 1. Other bias | | | Low risk | No other bias identified |
| ***Bellingan 2022*** | | | | |
| Methods | | Prospective, Phase 1/2, double‑blind, randomized, placebo‑controlled trial [The study enrolled 2 open-label, escalating dose tiers (cohorts 1 and 2), then selected the highest well-tolerated dose for a randomised, double-blind, placebo-controlled tier (cohort 3).]  Multicentric (12 centres) in UK and USA  Period of Inclusion: March 2016 and September 2018 | | |
| Participants | | **Inclusion criteria**  1. Male or female, age 18-90 years (inclusive);  2. Subject or legally authorized representative must freely sign informed consent after the nature of the trial and the disclosure of his/her data has been explained;  3. Diagnosis of a new acute onset of moderate to severe ARDS, as defined by the Berlin definition, requiring an endotracheal or tracheal tube with all the following criteria met, within a 24 hr period:  a. PaO2/FiO2 ratio of <200 mmHg (27 kPa) on positive end-expiratory airway pressure (PEEP) of ≥5 cm H2O;  b. Bilateral opacities on a chest radiograph or CT scan; and  c. Respiratory failure not fully explained by cardiac failure or fluid overload.  4. Able to receive investigational medicinal product within 96 hours of meeting the last of the ARDS diagnosis criterion defined in inclusion #3 (i.e., clock starts when last criterion is met);  5. Female subjects who are either:  a. Not pregnant, not breastfeeding, and are not planning on becoming pregnant for 3 months from IMP administration;  b. Not of childbearing potential, defined as one who has been postmenopausal for at least 1 year, or has been surgically sterilized, or has had a total hysterectomy at least 3 months prior to the start of this trial; or  c. If of childbearing potential, must agree to use an effective method of contraception for 3 months from IMP administration.  6. Male subjects with female partners of childbearing potential must agree to use adequate contraceptive methods (see Section Error! Reference source not found.) for 3 months from IMP administration.  **Exclusion criteria**  1. Moribund subject not expected to survive up to 48 hours;  2. Concurrent illness that shortens life expectancy to less than 6 months;  3. Home mechanical ventilation for chronic respiratory failure (Non-invasive ventilation (NIV) or via tracheostomy) except for Continuous Positive Airway Pressure (CPAP)/Bilevel positive airway pressure (BiPAP) used solely for sleep-disordered breathing;  4. Diffuse alveolar hemorrhage with or without vasculitis;  5. Severe ILD needing supplemental oxygen;  6. Severe COPD with recent FEV1/FVC ratio <0.3 (if available) or the use of home oxygen;  7. History of chronic pulmonary hypertension (WHO Class III or IV);  8. History of lung transplantation;  9. ST-segment elevation myocardial infarction (STEMI) within the last 6 months;  10. Mean arterial pressure (MAP) <60 mmHg while on 2 or more vasopressors with or without cardiovascular inotropic support;  11. Severe chronic liver disease (Childs-Pugh Score >10);  12. Known anaphylaxis or religious objection to bovine or porcine products;  13. Previous autologous, allogeneic bone marrow or peripheral stem cell transplant to treat conditions other than hematologic malignancies;  14. Any history of malignancy within the last 2 years, with the exception of adequately treated basal or squamous cell carcinoma of the skin or hematologic malignancy treated with bone marrow or peripheral stem cell transplantation;  15. History of human immunodeficiency virus (HIV) infection with the most recent CD4 T lymphocyte count, measured within the prior 6 months, being less than 200 cells/mm3 (testing of CD4 T lymphocyte count should be performed at screening for those subjects whose series of prior counts have been considered borderline for progressive disease, those for whom a count has not been performed within the prior 6 months, or on the recommendation of the Medical Monitor after consultation with the Investigator);  16. Clinical findings that, in the opinion of the Investigator, raise significant doubt that ARDS is the primary etiology of the subject’s hypoxemia and chest radiography criteria;  17. Other serious medical or psychiatric illness that, in the Investigator’s opinion, would not permit the subject to be managed according to the protocol;  18. Prior participation in any other clinical trial involving administration of a novel (unapproved) investigational pharmacological agent(s) within 30 days prior to enrollment. Concurrent enrollment in observational and device trials and trials involving administration of approved pharmacological agent(s) will be considered on a case by case basis and in consultation with the Medical Monitor; and  19. Significant sustained improvement in oxygenation following initial diagnosis of ARDS (meeting inclusion #3), suggesting resolving ARDS (P/F ratio > 300 mmHg (40 kPa)). P/F ratio should be confirmed ≤300 mmHg (40 kPa) within the 6 hours prior to randomization.  **Baseline characteristics**  N = 30, mean age of 55 years and 63.3% of men | | |
| Interventions | | Infusing 900 million cells (n = 20) or placebo (n = 10) within 96 h of ARDS diagnosis. | | |
| Outcomes | | **Primary endpoint**  Safety and tolerability of multipotent adult progenitor cells as assessed by (1) physiologic response within 4h of administration, monitoring vital signs, ventilator volumes and pressures, PaO2 or pulse oximetry, and ventilator setting or vasoactive medication dose adjustment, every 15min for the first 2h and at 3 and 4h after infusion start; and (2) occurrence of SUSARs within 24 h of administration.  **Secondary endpoints**  Assessment of vital signs and laboratory parameters through Day 28,  and TEAEs through Day 365.  Ventilator-free days  Days free from intensive care unit (ICU),  Total length of hospital stay through Day 28  Changes in PaO2/FiO2  Ratio and PEEP requirements from baseline through Days 1, 2, 3, 7 and 28  Changes in respiratory physiologic meas-  Ures (peak and plateau pressures) from baseline through  The time the subject is extubated; and all-cause mortality at Days 28, 90 and 365.  **Exploratory endpoints**  Changes in circulating biomarkers of inflammation and lung injury between baseline and Days 1, 2, 3 and 7; and health-related quality  of life (EQ-5D-3L) at Days 28, 90 and 365. | | |
| Notes | | 1. The study enrolled 2 open-label, escalating dose tiers (cohorts 1 and 2), then selected the highest well-tolerated dose for a randomised, double-blind, placebo-controlled tier (cohort 3). 2. Two of the authors, EJ and AT, were employees of Athersys and one, GB, received a travel grant from Athersys to two meetings | | |
| ***Cochrane Risk of bias*** | | | | |
| **Bias** | | | **Assessment** | **Support** |
| 1. Random sequence generation (selection bias) | | | Low risk | **Quote:** “Patients were randomised using an interactive web response system (Endpoint—Edinburgh, UK). ”  **Comment:** computer-generated random sequence is adequate. |
| 1. Allocation concealment (selection bias) | | | Low risk | **Quote:** “For each patient randomised, an unblinded staff member of the local cell processing facility received the patient treatment assignment and prepared the cell product or  placebo, placing a tinted cover over the intravenous infusion bag and tubing to conceal treatment allocation prior to dispensing.”  **Comment:** The method is adequate. |
| 1. Blinding of participants and personnel (performance bias) | | | Unclear risk | **Quote:** “Patients and all trial personnel, including investigators and clinicians, remained blinded to treatment assignment..”  **Comment:** No specific method was specified. |
| 1. Blinding of outcome assessment (detection bias) | | | Unclear risk | **Quote:** “Patients and all trial personnel, including investigators and clinicians, remained blinded to treatment assignment.”  **Comment:** No specific method was specified. |
| 1. Incomplete outcome data (attrition bias) | | | Low risk | **Quote:** “Lost to follow-up (n=0).”  **Comment:** It seems to be adequate. |
| 1. Selective reporting (reporting bias) | | | Low risk | **Comment:** in accordance with  the protocol available in clinical trials.gov. (NCT02611609) |
| 1. Other bias | | | High risk | 1. Baseline imbalance 2. Two of the authors, EJ and AT, were employees of Athersys and one, GB, received a travel grant from Athersys to two meetings |
| ***Lanzoni 2021*** | | | | |
| Methods | | Prospective, double-blind, phase 1/2a, randomized, controlled trial  Single centre in USA  Period of Inclusion: From 25 April 2020, to 21 July 2020 | | |
| Participants | | **Inclusion criteria**  Patient currently hospitalized  Aged ≥ 18 years  Willing and able to provide written informed consent, or with a legal representative who can provide informed consent  Peripheral capillary oxygen saturation (SpO2) ≤ 94% at room air, or requiring supplemental oxygen at screening  PaO2/FiO2 ratio < 300 mmHg  Bilateral infiltrates on frontal chest radiograph or bilateral ground glass opacities on a chest CT scan  **Exclusion criteria**  PaO2/FiO2 ≥ 300 at the time of enrollment  A previous MSC infusion not related to this trial  History of Pulmonary Hypertension (WHO Class III/IV)  History of left atrial hypertension or decompensated left heart failure.  Pregnant or lactating patient  Unstable arrhythmia  Patients with previous lung transplant  Patients currently receiving chronic dialysis  Patients currently receiving Extracorporeal Membrane Oxygenation (ECMO)  Presence of any active malignancy (except non-melanoma skin cancer)  Any other irreversible disease or condition for which 6-month mortality is estimated to be greater than 50%  Moderate to severe liver disease (AST and ALT >5 X ULN)  Severe chronic respiratory disease with a PaCO2 > 50 mm Hg or the use of home oxygen  Baseline QT prolongation  Moribund patient not expected to survive > 24 hours  **Baseline characteristics**  N = 24, mean age of 58.7 years and 54.2% of men | | |
| Interventions | | Subjects in the UC-MSC treatment group received two intravenous  infusions of 100 ± 20 × 106 UC-MSCs each, in 50 mL vehicle solution  containing human serum albumin and heparin, infused over 10 ± 5 minutes, at days 0 and 3. Subjects in the control group (n = 12) received two infusions of 50 mL vehicle solution, at day 0 and day 3. Best standard of care was provided in both groups following the current institutional COVID-19 guidelines. | | |
| Outcomes | | **Primary endpoint**  1. Safety, as defined by the occurrence of pre-specified infusion associated AEs, occurring within 6 hours from each infusion:  a. An increase in vasopressor dose greater than or equal to the following:  i. Norepinephrine: 10 μg per min  ii. Phenylephrine: 100 μg per min  iii. Dopamine: 10 μg/kg per min  iv. Epinephrine: 10 μg per min  b. In patients receiving mechanical ventilation: worsening hypoxemia, as assessed by a requirement for an increase of PEEP by 5 cm H2O over baseline, or requirement of a percentage increase in FiO2 of >20% from baseline  c. In patients receiving high flow oxygen therapy: worsening hypoxemia, as indicated by requirement of intubation and mechanical ventilation  d. New cardiac arrhythmia requiring cardioversion  e. New ventricular tachycardia, ventricular fibrillation, or asystole  f. A clinical scenario consistent with transfusion incompatibility or transfusion-related infection  2. Cardiac arrest or death within 24 h post infusion  3. Incidence of AEs.  **Secondary endpoints**  1. Survival at day 28  2. Time to recovery.  Time to recovery was defined as time to discharge or, if hospitalized, no longer requiring supplemental oxygen and no longer requiring COVID-19 related medical care. | | |
| ***Cochrane Risk of bias*** | | | | |
| **Bias** | | | **Assessment** | **Support** |
| 1. Random sequence generation (selection bias) | | | Low risk | **Quote:** “Twenty-four subjects hospitalized for COVID-19, confirmed positive for SARS-CoV-2 by PCR, were randomized 1:1 to either UC-MSC treatment (n=12) or Control group (n=12). Patients were assigned to treatment group using a stratified, blocked randomized design. Randomization tables were generated via SAS 9.4.”  **Comment:**  The method used to guarantee randomization seems to be adequate. |
| 1. Allocation concealment (selection bias) | | | Unclear risk | **Quote:** “The product label did not differentiate between UC-MSC or vehicle solution (control). The final product container was opaque.”  **Comment:** No specific method was specified. |
| 1. Blinding of participants and personnel (performance bias) | | | Unclear risk | **Quote:** “The study was double-blinded: neither the patient nor the assessing physician were aware of the treatment assignment, the staff responsible for product administration were blinded to group assignment.”  **Comment:** No specific method was specified. |
| 1. Blinding of outcome assessment (detection bias) | | | Unclear risk | **Quote:** “The study was double-blinded: neither the patient nor the assessing physician were aware of the treatment assignment, the staff responsible for product administration were blinded to group assignment. ”  **Comment:** No specific method was specified. |
| 1. Incomplete outcome data (attrition bias) | | | Low risk | **Quote:** “a total of 28 subjects were enrolled. Four subjects were subsequently determined to be ineligible because of screen failure. Twenty-four subjects were randomized (Figure 1). At enrollment, 11 subjects (46%) were receiving invasive mechanical ventilation, and 13 (54%) were on high flow oxygen therapy via noninvasive ventilation (including high flow nasal cannula, continuous positive airways pressure, or bilevel positive airways pressure) prior to initiation of treatment.”  **Comment:** It seems to be adequate. |
| 1. Selective reporting (reporting bias) | | | Low risk | **Comment:** in accordance with  the protocol available in clinical trials.gov. (NCT04355728) |
| 1. Other bias | | | Low risk | No other bias identified |
| ***Matthay 2019*** | | | | |
| Methods | | Prospective, double-blind, randomised, placebo-controlled, phase 2a trial  Multicentric (5 centres) and USA  Period of Inclusion: From March 24, 2014, to Feb 9, 2017 | | |
| Participants | | **Inclusion criteria**  Eligible patients were endotracheally intubated, had a PaO2:FiO2 less than 27 kPa, were mechanically ventilated with at least 8 cm H2O positive-end expiratory pressure (PEEP), had bilateral pulmonary infiltrates consistent with pulmonary oedema on chest radiographs, and had no clinical evidence of left-heart failure or volume overload as the primary cause of the pulmonary oedema. A protocol amendment was made to allow enrolment of patients with PEEP of  5 cm H2O if they had evidence of barotrauma.  **Exclusion criteria**  age younger than 18 years, ARDS present for more than 96 h, pregnancy or breastfeeding, being an inmate in the prison system, having received treatment for cancer in the past 2 years (except non-melanoma skin cancer), having an underlying medical status with life expectancy less than 6 months, moderate to severe liver disease  (Child-Pugh score >12), severe chronic lung disease with the use of home oxygen, or partial arterial pressure of carbon dioxide greater than 7 kPa, and not being committed to full support (ie, had do not resuscitate or limit on life support orders).  **Baseline characteristics**  N = 60, mean age of 55 years and 55% of men | | |
| Interventions | | Biological: Allogeneic Bone Marrow-Derived Human Mesenchymal Stromal Cells; 10 × 10⁶ MSC/kg  Allogeneic Bone Marrow-Derived Human Mesenchymal Stromal Cells was administered intravenously over approximately 60-80 minutes.  Biological: Plasma-Lyte A; 10 × 10⁶*placebo*/kg  Plasma-Lyte A placebo was administered intravenously over approximately 60-80 minutes. | | |
| Outcomes | | **Primary endpoint**  The safety of the MSC infusion, assessed with prespecified infusion-associated adverse events focused on acute haemo dynamic or respiratory compromise.  **Secondary endpoints**  All-cause mortality at day 28 and day 60, number of ventilator-free days to day 28, duration of ventilation in patients alive at day 28, number of intensive-care-free days to day 28, number of days free from organ failure to day 28 (cardiovascular, coagulation, hepatic, and renal), and the sequential organ failure assessment (SOFA) score. | | |
| ***Cochrane Risk of bias*** | | | | |
| **Bias** | | | **Assessment** | **Support** |
| 1. Random sequence generation (selection bias) | | | Low risk | **Quote:** “Patients were randomly assigned (2:1) to receive either 10 ×10^6^MSC/kg predicted bodyweight or placebo (PlasmaLyte A, Baxter, Deerfield, IL, USA). The randomisation had a variable block design, was stratified by site, and the sequence was generated by computer.”  **Comment:** The method used to guarantee randomization seems to be adequate. |
| 1. Allocation concealment (selection bias) | | | Low risk | **Quote:** “ The allocation sequence could be accessed by each cell laboratory through a dedicated website. Personnel in the cell laboratories were not masked, but patients, clinical staff, and investigators were unaware of treatment assignment. bags containing the study products and intravenous tubing had opaque coverings applied in the cell laboratories. .”  **Comment:** The method is adequate. |
| 1. Blinding of participants and personnel (performance bias) | | | Unclear risk | **Quote:** “ To maintain masking of the investigators and clinicians, bags containing the study products and intravenous tubing had opaque coverings applied in the cell laboratories. No measures were taken to check the success of masking. For data analysis, investigators were unaware of treatment allocation until the database was fully cleaned and locked.”  **Comment:** No specific method was specified. |
| 1. Blinding of outcome assessment (detection bias) | | | Unclear risk | **Quote:** “double-blinded”  **Comment:** No specific method was specified. |
| 1. Incomplete outcome data (attrition bias) | | | Low risk | **Quote:** “1038 patients were screened for eligibility, of whom 975 were excluded, and 63 were randomly assigned to a treatment group. Three patients were not eligible for treatment  after randomisation and, therefore, 40 patients received MSCs and 20 patients received placebo”  **Comment:** It seems to be adequate. |
| 1. Selective reporting (reporting bias) | | | Low risk | **Comment:** in accordance with  the protocol available in clinical trials.gov. (NCT02097641) |
| 1. Other bias | | | High risk | There was baseline imbalance; there are interests between researchers. |
| ***Zheng 2014*** | | | | |
| Methods | | Prospective, randomized, double-blind, and placebo-controlled study.  Single center in China  Period of Inclusion: Between January and April 2013 | | |
| Participants | | **Inclusion criteria**  at least 18 years of age and diagnosed within 48 hours with a PaO2/FiO2 ratio of < 200.  **Exclusion criteria**  pre-existing severe disease of any major organs, pregnancy, pulmonary hypertension, malignant disease, human immunodeficiency virus (HIV) infection or if informed consent could not be obtained.  **Baseline characteristics**  N = 12, mean age of 68.25 years and 91.7% of men | | |
| Interventions | | Patients were randomized upon study enrollment. For all patients, a negative fluid balance was maintained by diuretics and fluid restriction. ARDS Network low tidal volume protocol was adopted for standardized ventilator management, targeting a tidal volume of 8 ml/kg of the patient’s ideal body weight and a plateau pressure less  than 30 mmHg [20]. Per the requirement of Research Ethics Committee at Shaoxing Second Hospital, frozen MSCs with DMSO and fetal bovine serum were not allowed to be infused to patients directly. For the MSCs group, frozen cells were immediately thawed, cultured with patient’s own serum and harvested in 24-48 hours. Freshly harvested MSCs, at a dose of 1 × 106 cells/kg body weight, were suspended in 100 ml normal saline for peripheral intravenous infusion and administered over 1 hour within 48 hours of enrollment. For the placebo group, a bag of 100 ml normal saline was infused at similar time point. | | |
| Outcomes | | **Primary endpoint**  The occurrence of adverse events.  **Secondary endpoints**  the following: PaO2/FiO2 ratio  hospital indices (length of hospital stay, ventilator-free days and ICU-free days at day 28)  serum biomarkers of ARDS including IL-6, IL-8 and SP-D. | | |
| ***Cochrane Risk of bias*** | | | | |
| **Bias** | | | **Assessment** | **Support** |
| 1. Random sequence generation (selection bias) | | | Unclear risk | **Quote:** “a randomized trail”  **Comment:** No specific method was specified. |
| 1. Allocation concealment (selection bias) | | | Unclear risk | No specific method was specified. |
| 1. Blinding of participants and personnel (performance bias) | | | Unclear risk | **Quote:** “double-blind”  **Comment:** No specific method was specified. |
| 1. Blinding of outcome assessment (detection bias) | | | Unclear risk | **Quote:** “double-blinded”  **Comment:** No specific method was specified. |
| 1. Incomplete outcome data (attrition bias) | | | Low risk | **Quote:** “During the study period,  one patient in the MSCs group died of multiple organ failure. Deaths occurred in two patients in the placebo group with one multiple organ failure and the other sepsis.None of the deaths were considered to be related to the study drugs by the clinical investigators and were consistent with the patients’ existing disease processes.”  **Comment:** It seems to be adequate. |
| 1. Selective reporting (reporting bias) | | | Low risk | **Comment:** in accordance with  the protocol available in clinical trials.gov. (NCT01902082) |
| 1. Other bias | | | Low risk | No other bias identified |
