# Supplementary material for: Safety, efficacy and biomarkers analysis of mesenchymal stromal cells therapy in ARDS: a systematic review and meta-analysis based on phase I and II RCTs
Source: Stem Cell Res Ther. 2022 Jun 25;13:275. doi: 10.1186/s13287-022-02956-3 (PMC9233855; doi:10.1186/s13287-022-02956-3)
Supplement: Supplementary file 5 — Additional file 5. Quality of GRADE. [file 13287_2022_2956_MOESM5_ESM.docx]

**Supplementary Table 5 Quality of GRADE**

| **Quality assessment** | | | | | | | **No of patients** | | **Effect** | | **Quality** | **Importance** |  |
| --- | --- | --- | --- | --- | --- | --- | --- | --- | --- | --- | --- | --- | --- |
|  |  |  |  |  |  |  |  |  |  |  |  |  |  |
| **No of studies** | **Design** | **Risk of bias** | **Inconsistency** | **Indirectness** | **Imprecision** | **Other considerations** | **MSCs** | **Placebo** | **Relative (95% CI)** | **Absolute** |  |  |  |
| **Subjects with AEs and SAEs - Subjects with AEs** | | | | | | | | | | | | |  |
| 4 | randomised trials | serious^1^ | serious | no serious indirectness | serious^2^ | none | 46/59  (78%) | 36/52  (69.2%) | OR 1.68 (0.43 to 6.6) | 98 more per 1000 (from 201 fewer to 245 more) | ⊕OOO VERY LOW | CRITICAL |  |
|  |  |  |  |  |  |  |  | 67.5% |  | 102 more per 1000 (from 203 fewer to 257 more) |  |  |  |
| **Subjects with AEs and SAEs - Subjects with SAEs** | | | | | | | | | | | | |  |
| 3 | randomised trials | serious^3^ | serious^4^ | no serious indirectness | serious^5^ | none | 20/53  (37.7%) | 20/46  (43.5%) | OR 0.57 (0.14 to 2.32) | 130 fewer per 1000 (from 338 fewer to 206 more) | ⊕OOO VERY LOW | CRITICAL |  |
|  |  |  |  |  |  |  |  | 60% |  | 139 fewer per 1000 (from 426 fewer to 177 more) |  |  |  |
| **Subjects with AEs and SAEs - Lanzoni's SAEs** | | | | | | | | | | | | |  |
| 1 | randomised trials | serious^6^ | no serious inconsistency | no serious indirectness | no serious imprecision | none | 2/12  (16.7%) | 8/12  (66.7%) | OR 0.1 (0.01 to 0.69) | 500 fewer per 1000 (from 87 fewer to 647 fewer) | ⊕⊕⊕O MODERATE | CRITICAL |  |
|  |  |  |  |  |  |  |  | 66.7% |  | 500 fewer per 1000 (from 87 fewer to 647 fewer) |  |  |  |
| **Mortality analysis - D28 mortality** | | | | | | | | | | | | |  |
| 5 | randomised trials | serious^7^ | serious^8^ | no serious indirectness | serious^9^ | none | 24/98  (24.5%) | 18/72  (25%) | OR 0.93 (0.45 to 1.89) | 13 fewer per 1000 (from 120 fewer to 137 more) | ⊕OOO VERY LOW | CRITICAL |  |
|  |  |  |  |  |  |  |  | 33.3% |  | 16 fewer per 1000 (from 150 fewer to 152 more) |  |  |  |
| **Mortality analysis - D60 mortality** | | | | | | | | | | | | |  |
| 1 | randomised trials | serious^10^ | no serious inconsistency | no serious indirectness | serious^11^ | none | 15/40  (37.5%) | 5/20  (25%) | OR 1.8 (0.54 to 5.96) | 125 more per 1000 (from 97 fewer to 415 more) | ⊕⊕OO LOW | CRITICAL |  |
|  |  |  |  |  |  |  |  | 25% |  | 125 more per 1000 (from 97 fewer to 415 more) |  |  |  |
| **Mortality analysis - 1 year mortality** | | | | | | | | | | | | |  |
| 1 | randomised trials | serious^12^ | no serious inconsistency | no serious indirectness | serious^13^ | none | 8/20  (40%) | 5/10  (50%) | OR 0.67 (0.14 to 3.07) | 99 fewer per 1000 (from 377 fewer to 254 more) | ⊕⊕OO LOW | CRITICAL |  |
|  |  |  |  |  |  |  |  | 50% |  | 99 fewer per 1000 (from 377 fewer to 254 more) |  |  |  |
| **Mortality analysis - Subjects with PaO2/FiO2 < 150 mmHg, D28 mortality** | | | | | | | | | | | | |  |
| 1 | randomised trials | serious^14^ | no serious inconsistency | no serious indirectness | serious^15^ | none | 2/8  (25%) | 4/8  (50%) | OR 0.33 (0.04 to 2.77) | 252 fewer per 1000 (from 462 fewer to 235 more) | ⊕⊕OO LOW | CRITICAL |  |
|  |  |  |  |  |  |  |  | 50% |  | 252 fewer per 1000 (from 462 fewer to 235 more) |  |  |  |

^1^ Bellingan's study blind method was not described in detail and baseline data were imbalanced; The study randomization method, allocation scheme, and blind method in Zheng were not described in detail and could not be assessed; Lanzoni's study assignment protocol and blind method were not described in detail and could not be assessed.
^2^ Small sample size
^3^ Bellingan's study blind method was not described in detail and baseline data were imbalanced; Lanzoni's study assignment protocol and blind method were not described in detail and could not be assessed.
^4^ Belligan and Mosel's OR ≥1 while Lanzoni's OR < 1
^5^ Small sample size
^6^ Lanzoni's study assignment protocol and blind method were not described in detail and could not be assessed.
^7^ Bellingan's study blind method was not described in detail and baseline data were imbalanced. The study randomization method, allocation scheme, and blind method in Zheng were not described in detail and could not be assessed. Lanzoni's study assignment protocol and blind method were not described in detail and could not be assessed; Matthay's study blind method was not described in detail and could not be assessed.
^8^ Some studies had an OR > 1.
^9^ Small sample size.
^10^ Blind method not specified.
^11^ Small sample size
^12^ Blind method not specified.
^13^ Small sample size
^14^ Blind method not specified.
^15^ Small sample size
