# Supplementary material for: Safety, efficacy and biomarkers analysis of mesenchymal stromal cells therapy in ARDS: a systematic review and meta-analysis based on phase I and II RCTs
Source: Stem Cell Res Ther. 2022 Jun 25;13:275. doi: 10.1186/s13287-022-02956-3 (PMC9233855; doi:10.1186/s13287-022-02956-3)
Supplement: Supplementary file 6 — Additional file 6. Summary of Findings. [file 13287_2022_2956_MOESM6_ESM.docx]

| **Supplementary Table 6 Summary of Findings table ( MSCs compared to Placebo for ARDS)** | | | | | | |
| --- | --- | --- | --- | --- | --- | --- |
| **Patient or population:** patients with ARDS **Settings:** in-hospital **Intervention:** MSCs **Comparison:** Placebo | | | | | | |
| **Outcomes** | **Illustrative comparative risks* (95% CI)** | | **Relative effect (95% CI)** | **No of Participants (studies)** | **Quality of the evidence (GRADE)** | **Comments** |
|  | Assumed risk | Corresponding risk |  |  |  |  |
|  | **Placebo** | **MSCs** |  |  |  |  |
| **Subjects with AEs and SAEs - Subjects with AEs** | **Study population** | | **OR 1.68**  (0.43 to 6.6) | 111 (4 studies) | ⊕⊝⊝⊝ **very low**^1,2^ |  |
|  | **692 per 1000** | **791 per 1000** (492 to 937) |  |  |  |  |
|  | **Moderate** | |  |  |  |  |
|  | **675 per 1000** | **777 per 1000** (472 to 932) |  |  |  |  |
| **Subjects with AEs and SAEs - Subjects with SAEs** | **Study population** | | **OR 0.57**  (0.14 to 2.32) | 99 (3 studies) | ⊕⊝⊝⊝ **very low**^3,4,5^ |  |
|  | **435 per 1000** | **305 per 1000** (97 to 641) |  |  |  |  |
|  | **Moderate** | |  |  |  |  |
|  | **600 per 1000** | **461 per 1000** (174 to 777) |  |  |  |  |
| **Subjects with AEs and SAEs - Lanzoni's SAEs** | **Study population** | | **OR 0.1**  (0.01 to 0.69) | 24 (1 study) | ⊕⊕⊕⊝ **moderate**^6^ |  |
|  | **667 per 1000** | **167 per 1000** (20 to 580) |  |  |  |  |
|  | **Moderate** | |  |  |  |  |
|  | **667 per 1000** | **167 per 1000** (20 to 580) |  |  |  |  |
| **Mortality analysis - D28 mortality** | **Study population** | | **OR 0.93**  (0.45 to 1.89) | 170 (5 studies) | ⊕⊝⊝⊝ **very low**^7,8,9^ |  |
|  | **250 per 1000** | **237 per 1000** (130 to 387) |  |  |  |  |
|  | **Moderate** | |  |  |  |  |
|  | **333 per 1000** | **317 per 1000** (183 to 485) |  |  |  |  |
| **Mortality analysis - D60 mortality** | **Study population** | | **OR 1.8**  (0.54 to 5.96) | 60 (1 study) | ⊕⊕⊝⊝ **low**^10,11^ |  |
|  | **250 per 1000** | **375 per 1000** (153 to 665) |  |  |  |  |
|  | **Moderate** | |  |  |  |  |
|  | **250 per 1000** | **375 per 1000** (153 to 665) |  |  |  |  |
| **Mortality analysis - 1 year mortality** | **Study population** | | **OR 0.67**  (0.14 to 3.07) | 30 (1 study) | ⊕⊕⊝⊝ **low**^12,13^ |  |
|  | **500 per 1000** | **401 per 1000** (123 to 754) |  |  |  |  |
|  | **Moderate** | |  |  |  |  |
|  | **500 per 1000** | **401 per 1000** (123 to 754) |  |  |  |  |
| **Mortality analysis - Subjects with PaO2/FiO2 < 150 mmHg, D28 mortality** | **Study population** | | **OR 0.33**  (0.04 to 2.77) | 16 (1 study) | ⊕⊕⊝⊝ **low**^14,15^ |  |
|  | **500 per 1000** | **248 per 1000** (38 to 735) |  |  |  |  |
|  | **Moderate** | |  |  |  |  |
|  | **500 per 1000** | **248 per 1000** (38 to 735) |  |  |  |  |
| *The basis for the **assumed risk** (e.g. the median control group risk across studies) is provided in footnotes. The **corresponding risk** (and its 95% confidence interval) is based on the assumed risk in the comparison group and the **relative effect** of the intervention (and its 95% CI). **CI:** Confidence interval; **OR:** Odds ratio; | | | | | | |
| GRADE Working Group grades of evidence **High quality:** Further research is very unlikely to change our confidence in the estimate of effect.  **Moderate quality:** Further research is likely to have an important impact on our confidence in the estimate of effect and may change the estimate. **Low quality:** Further research is very likely to have an important impact on our confidence in the estimate of effect and is likely to change the estimate. **Very low quality:** We are very uncertain about the estimate. | | | | | | |
| ^1^ Bellingan's study blind method was not described in detail and baseline data were imbalanced; The study randomization method, allocation scheme, and blind method in Zheng were not described in detail and could not be assessed; Lanzoni's study assignment protocol and blind method were not described in detail and could not be assessed. ^2^ Small sample size ^3^ Bellingan's study blind method was not described in detail and baseline data were imbalanced; Lanzoni's study assignment protocol and blind method were not described in detail and could not be assessed.  ^4^ Belligan and Mosel's OR ≥1 while Lanzoni's OR < 1 ^5^ Small sample size ^6^ Lanzoni's study assignment protocol and blind method were not described in detail and could not be assessed.  ^7^ Bellingan's study blind method was not described in detail and baseline data were imbalanced. The study randomization method, allocation scheme, and blind method in Zheng were not described in detail and could not be assessed. Lanzoni's study assignment protocol and blind method were not described in detail and could not be assessed; Matthay's study blind method was not described in detail and could not be assessed. ^8^ Some studies had an OR > 1. ^9^ Small sample size. ^10^ Blind method not specified. ^11^ Small sample size ^12^ Blind method not specified. ^13^ Small sample size ^14^ Blind method not specified. ^15^ Small sample size | | | | | | |
| Note: Because of the scatter in reported oxygenation index and biomarker data across studies, statistical assessment was not possible, but overall the quality of evidence was low. | | | | | | |
